# Manometry of the Human Ileum and Ileocaecal Junction in Health, Disease and Surgery: A Systematic Review

**DOI:** 10.3389/fsurg.2020.00018

**Published:** 2020-04-15

**Authors:** Chen Liu, Kai Sheng Saw, Phil G. Dinning, Gregory O'Grady, Ian Bissett

**Affiliations:** ^1^Department of Surgery, University of Auckland, Auckland, New Zealand; ^2^Departments of Gastroenterology and Surgery, Flinders Medical Centre, Flinders University, Adelaide, SA, Australia

**Keywords:** manometry, ileum, terminal ileum, ileocaecal junction, motility, small bowel

## Abstract

**Background:** The terminal ileum and ileocaecal junction form a transition zone in a relatively inaccessible portion of the gastrointestinal tract. Little is known about the motility of this region with few detailed studies, indicating the need for a robust synthesis of current knowledge. This review aimed to evaluate the quantitative and qualitative data on the manometry findings of the terminal ileum and ileocaecal junction during the fasting and post-prandial periods in healthy individuals and patients with motility disorders or patients after bowel surgery.

**Methods:** A systematic search of five databases (Medline, Pubmed, Embase, Scopus, and Cochrane Library) was performed. Studies that presented manometry data from the human ileum or ileocaecal junction were included.

**Results:** Forty-two studies met the inclusion criteria. The main motility patterns reported in the terminal ileum during fasting were the migrating motor complex, discrete clustered contractions, prolonged propagated contractions and phasic contractions. Post-prandial motility featured irregular, intense contractions. Some studies found a region of sustained increased pressure at the ileocaecal junction while others did not. Patients with motility disorders showed differences in manometry including retrograde propagation of phase III. Patients post-bowel surgery showed differences including higher incidence of phase III.

**Conclusion:** Motility patterns of the terminal ileum differ between fasting and fed states. Large variability existed in manometry recordings of the terminal ileum. Technical challenges and lack of standardized definitions may reduce accuracy of manometry assessment. Further research is needed to understand how this key portion of the gut physiologically functions.

## Introduction

### Rationale

The terminal ileum is an important junctional zone with specialized functions. This region of the small bowel is involved in the active absorption of bile acids and vitamin B12 (1, 2), and the ileocaecal junction (ICJ) helps to coordinate efflux of contents to the colon while preventing coloileal reflux (3, 4). These functions help to maintain gut homeostasis by helping to regulate the transit of fluid into the colon to a volume that is within its absorptive capacity (~1.5 L of chyme per day) (5), while also separating the dense microbiota of the large bowel from the ileum (6).

Despite its importance, accurate assessment of the motility profile of the ileum in humans has been limited. Unlike the ileum, there is an abundance of manometry studies involving both the upper gastrointestinal tract (including jejunum) and the large bowel. The terminal ileum has received less attention, likely due to its relative inaccessibility (7).

Obtaining accurate motility information from the terminal ileum has important clinical implications, by informing normal physiology and pathophysiology. For example, disturbances of the Migrating Motor Complex (MMC) pattern have been proposed in the proximal small bowel of patients with a variety of gastrointestinal disorders, ranging from bacterial overgrowth and systemic sclerosis to myotonic dystrophy and chronic idiopathic intestinal pseudo-obstruction (8–11). To date, only a small number of studies have focused on ileal manometry abnormalities in motility disorders such as irritable bowel syndrome (IBS) and chronic idiopathic constipation (12, 13).

There have been no attempts in the literature to consolidate and evaluate the available data pertaining to manometry of the terminal ileum in the context of health, motility disorders or bowel surgery. Performing such a review would consolidate and synthesize the knowledge researchers have gained thus far, and also highlight limitations and direct future areas of investigation.

### Objectives and Research Questions

This systematic review therefore had three key objectives:

1. Evaluate the quantitative and qualitative data on the manometry findings of the terminal ileum and ileocaecal junction in normal healthy human subjects during the interdigestive and post-prandial periods.

2. Evaluate known manometry findings of the terminal ileum in patients with motility disorders.

3. Evaluate known manometry findings of the terminal ileum in patients who have undergone bowel surgery, such as loop ileostomy.

## Methods

### Study Design

The systematic review was conducted in accordance with the checklist of reporting items within the PRISMA guidelines (14).

### Systematic Review Protocol

A systematic literature search of the Ovid Medline, Pubmed, Embase, Scopus, and Cochrane Library databases was performed. All studies that featured original research in which manometry data were collected from the terminal ileum were included. Exclusion criteria were studies that did not specifically describe data from the ileum, review articles, letters to the editor, case reports and conference abstracts. Studies that focused on ileal pouches following proctocolectomy were only included if manometry data was also collected proximal to the pouch.

Searches were performed concurrently and independently by two authors (CL and KS). Titles and abstracts were screened based on the above criteria. Full texts were then reviewed to confirm eligibility. In cases where there was insufficient information from title and abstract alone, full texts were obtained and screened. Group consensus was required for study inclusion and any disputes were adjudicated by a senior author (IB).

### Search Strategy

An example of the search strategy used is as follows: [“ileum” (keywords and mesh) OR “ileocaecal valve (keywords and mesh)] AND [“manometry” (keywords and mesh) or “manometer^*^” (keyword)]. Studies were restricted to human subjects and English language. No limits were applied to the time of publication or age of participants. The reference bibliographies of all included articles were hand searched to identify any other relevant studies.

### Data Extraction

Data were extracted by a single author (CL) into a summary table and the accuracy was then checked by a second author (KS). Attempts were made to contact the study author via e-mail if the reported data contained a suspected error or a major discrepancy.

Details of the study design were collected including type of participant (i.e., healthy volunteers vs. motility disorder patients vs. bowel surgery patients) and the duration of manometry recording (fasting and post-prandial). Specifications of the manometry recording technique were also collected, such as the type of manometry catheter, the number of sensors and inter-sensor spacing.

Quantitative information regarding the pressure profiles of motility events were collected, including the incidence of the event, contraction frequency, amplitude, duration, and propagation velocity and distance. Qualitative information was also collected, including authors' definitions of various motility phenomena, descriptions of motility events, and comments on the strengths and weaknesses of their manometry technique.

### Data Analysis

Quantitative data related to healthy patients were analyzed separately to data from patients with motility disorders, which in turn were analyzed separately from patients after bowel surgery. It became apparent after this categorization that there were often very few data points per analyzed parameter. In addition, there was significant heterogeneity of motility pattern definitions adopted by different studies. For these reasons, no quantitative meta-analysis was performed. Summary descriptive statistics were instead formulated and presented for each parameter, with a narrative synthesis of overall findings.

No specific bias evaluation tools or scores were employed given the significant heterogeneity of study designs, manometry recording techniques and participant populations. The potential limitations pertaining to the methodology of each study were evaluated and highlighted in a summary table.

## Results

### Flow Diagram

**Figure 1:**
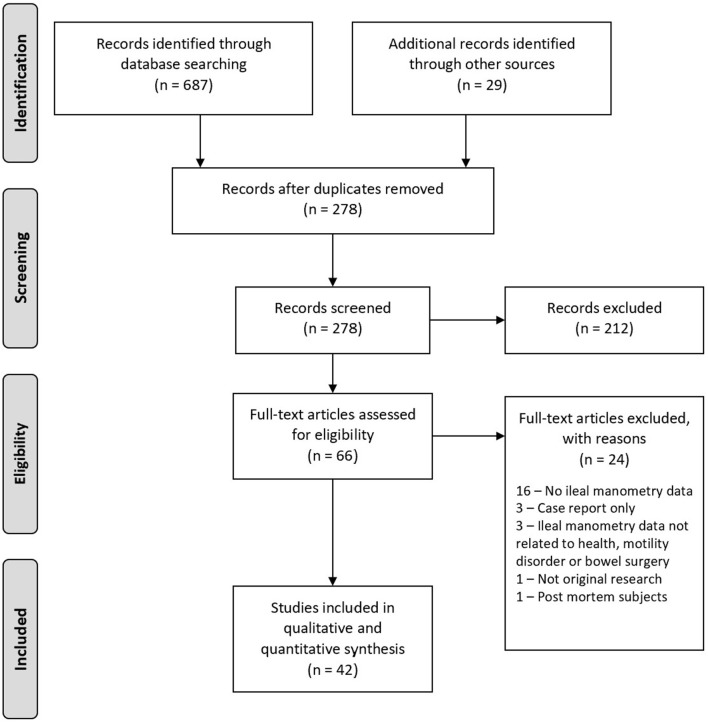
PRISMA flow diagram.

The PRISMA flow diagram (14) is presented in Figure 1, including the reasons for exclusion following full text review.

### Study Selection and Characteristics

The initial search found a total of 687 articles. After exclusion of duplicates and initial screening of titles and abstracts, 66 articles underwent full text review, from which 42 original research articles were included.

The characteristics of the included studies are presented in Table 1. Twenty-eight studies contained data on healthy volunteers, five studies featured motility disorder patients, and 16 studies featured bowel surgery patients, including 10 studies that used an ileostomy as a manometry access point. One study involved a pediatric population (47).

**Table 1:** Details of included articles.

**References**	**Study population**	**Study size (no. of patients)**	**Duration of recording (h)**		**Type of manometry assembly**	**No. of sensors in TI**	**Spacing between sensors (cm)**	**Limitations**
			**Fasting**	**Post-prandial**				
Accarino et al. (15)	Salmonella patients and healthy volunteers	22 (12 healthy, 10 with Salmonella)	6	0	Perfused catheter	4	20	No post-prandial recording, large sensor spacing
Barberani et al. (16)	Ileostomy patients and healthy volunteers undergoing colonoscopy	29 (nine with ileostomy, 20 healthy)	5–20 min	0	Perfused catheter	3–4	0.4–5	Short recording duration, no post-prandial recording, need for bowel prep
Bassotti et al. (17)	Proctocolectomy patients with J-shaped Ileal Reservoir	6	1.5	1	Perfused catheter	two in ileum, two in pouch	5	Short recording duration, limited number of sensors
Borody et al. (18)	Healthy volunteers	16	6–8	5	Perfused catheter	9	1.5–20	Large sensor spacing for some sensors
Castell et al. (19)	Colonic bypass patients	3	Unknown	Unknown	Perfused catheter	3	5	Unknown duration of recording
Code et al. (20)	Ileostomy patients	2	19–28.5	1	Photokymograph with tandem balloons	2	3	Small study size, limited number of sensors
Coffin et al. (21)	Healthy volunteers	10	1	2	Perfused catheter	3	5–10	Short recording duration, large sensor spacing
Cohen et al. (3)	Colonic exclusion and colostomy patients	5	<1	0	Perfused catheter	1	N/A	Short recording duration, only one sensor in TI, no post-prandial recording
Corazziari et al. (22)	Healthy volunteers undergoing colonoscopy	11	<1	0	Perfused catheter	3	0.4	Short recording duration, need for bowel prep
Cummins (23)	Ileostomy patients	5	Unknown	Unknown	Kymograph with rubber balloon	1	N/A	Unknown recording duration, only one sensor
Daniel et al. (24)	Ileostomy patients	4	0.5	1.5–3.5	Perfused catheter with balloon	1	N/A	Short fasting recording duration, only one sensor
Dinning et al. (25)	Healthy volunteers	14	24 (with three meals during this period)		Perfused catheter (silastic)	5	7.5	Large sensor spacing
Dinning et al. (4)	Ileostomy patients	10	2	2	Perfused catheter (silicon)	6	5–7.5	Short recording period, large sensor spacing
Dinning et al. (26)	Healthy volunteers	6	1	3	Perfused catheter (silastic)	Unknown	7.5	Short recording period, large sensor spacing, number of sensors in TI unknown
Dinning et al. (27)	Healthy volunteers	6	0	4–6	Perfused catheter (silicon)	≥4	7.5	No fasting recording, large sensor spacing
Gorard et al. (28)	IBS patients and healthy volunteers	20 (eight with IBS, 12 healthy)	~17 per patient	0	Solid state catheter	1	N/A	No post-prandial recording, only one sensor in TI
Groom et al. (29)	Proctocolectomy patients with ileoanal pouch	12	24 (patient able to eat as usual)		Solid state catheter	5	20	Large sensor spacing
Hammer et al. (30)	Healthy volunteers	13	1	~4	Perfused catheter	3–4	10–20	Short fasting recording duration, large sensor spacing
Kachel et al. (31)	Healthy volunteers	9	~6.5	0	Perfused catheter	2	50	No post-prandial recording, limited number of sensors, large sensor spacing
Kamath et al. (32)	Healthy volunteers	23	7	0	Perfused catheter	3	Unknown	No post-prandial recording, unknown sensor spacing
Kellow et al. (33)	Healthy volunteers	16	15–29	1.5–4	Perfused catheter	4	10–15	Large sensor spacing
Kellow et al. (34)	Healthy volunteers	8	N/A	3	Perfused catheter	3–6	10–20	No fasting recording, large sensor spacing
Kellow et al. (35)	IBS patients and healthy volunteers	32 (16 with IBS, 16 healthy)	12–29	Mean 1.8–2.2	Perfused catheter	4	Unknown	Unknown sensor spacing
Kellow et al. (12)	IBS patients and healthy volunteers	24 (16 with IBS, eight healthy)	Overnight	3	Perfused catheter	3–6	Unknown	Unknown sensor spacing
Kerlin et al. (7)	Healthy volunteers	11	6	6	Perfused catheter	Unknown	20	Unknown sensor number
Kerlin et al. (36)	Healthy volunteers	10	6	6	Perfused catheter	Unknown	20	Unknown sensor number
Kerlin et al. (37)	Healthy volunteers	6	24 (with three meals during this period)		Perfused catheter	1–2	40	Limited number of sensors, large sensor spacing
Luiking et al. (38)	Healthy volunteers	8	~8.5	0	Perfused catheter (silicon)	5	12.5	No post-prandial recording, large sensor spacing
Miedema et al. (39)	Proctocolectomy with ileal pouch-anal anastomosis and loop ileostomy	8	3	3	Perfused catheter	3	5	Short recording period, limited number of sensors
Miedema et al. (40)	Proctcolectomy with ileal pouch-anal anastomosis and loop ileostomy	13	3	3	Perfused catheter	3	5	Short recording period, limited number of sensors
Nasmyth et al. (41)	Ileostomy patients	7	Unknown	Unknown	Perfused catheter	2	6–8	Unknown duration of recording, limited number of sensors, large sensor spacing
Panagamuwa et al. (13)	Chronic idiopathic constipation patients and healthy volunteers	16 (10 with constipation, six healthy)	24 (patient able to eat as usual)		Silicone-coated catheter	2	15	Limited number of sensors, large sensor spacing
Penagini et al. (42)	Healthy volunteers	10	0	5.5	Perfused catheter	3–5	7.5	No fasting recording, large sensor spacing
Pescatori (43)	Proctocolectomy with ileoanal reservoir	7	3	1	Perfused catheter	3	Unknown	Short recording duration, limited number of sensors, unknown sensor spacing
Posey and Bargen (44)	Ileostomy patients	10	0	1–3	Photokymograph with tandem balloons	2	Unknown	No fasting recording, short post-prandial recording duration, limited number of sensors, unknown sensor spacing
Quigley et al. (45)	Healthy volunteers	22	6–22	12	Perfused catheter	~8	1.5–25	Some large sensor spacing
Seidl et al. (46)	Healthy volunteers	10	24 (with one standardized meal)		Polyurethane catheter with piezoresistive pressure sensors	Unknown	3	Unknown number of sensors in TI
Sood et al. (47)	Ileostomy patients and endorectal pull-through patients	29 (23 with ileostomy, six with endorectal pull-through)	1–4	1–4	Perfused catheter	4–8	5–15	Variable duration of recording, large sensor spacing
Spiller et al. (48)	Healthy volunteers	13	Overnight	5	Perfused catheter	6	4–10	Some large sensor spacing
Spiller et al. (49)	Healthy volunteers	15	Overnight	2	Perfused catheter	7–8	4–10	Some large sensor spacing
Stryker et al. (50)	Proctocolectomy patients with ileal pouch-anal anastomosis and healthy volunteers	14 (eight with proctocolectomy and 6 healthy)	16–23	6	Perfused catheter	Unknown	10–25	Unknown number of sensors in TI, large sensor spacing
Van Ooteghem et al. (51)	Healthy volunteers	8	Unknown	0	Perfused catheter (silicon)	8	10	Unknown duration of fasting recording, no post-prandial recording, large sensor spacing

### Synthesized Findings

#### Definitions of Motility Patterns

The most common ileal motility patterns reported were the migrating motor complex (MMC) and its three phases (I, II, and III), discrete clustered contractions (DCC), prolonged propagated contractions (PPC) and phasic contractions. Significant variability existed in the definitions of these terms. There was variability in the definitions' levels of detail. For example, five studies defined “Phase I” of the MMC as simply a period of motor quiescence (29, 36, 46, 47, 51). However, one study characterized it as a 10-min period with no more than 3 single contractions greater than 15 mmHg (28), while another described it as a period of motor quiescence except for occasionally occurring isolated pressure peaks over a period of <3 min (31).

Variability also existed in the quantitative criteria of each definition. Prolonged propagated contractions were described by most studies as a single wave with large amplitude and long duration but the minimum amplitude requirement ranged from 20 to 60 mmHg (32, 40) while the minimum duration requirement ranged from 6 to 12 s (40, 46). In view of this variability, summary definitions were derived and displayed in Table 2. The definitions shown are a combination of the most common and more inclusive criteria observed in the published studies.

**Table 2:** Summary definitions of the most common ileal manometry motility terms.

**Ileal motility pattern**		**Pattern similar to…**	**No. of studies with definition (References)**	**Summary definition**
MMC	Phase I		8 (7, 12, 15, 28, 29, 31, 46, 51)	Motor quiescence
	Phase II		6 (7, 13, 15, 29, 31, 46, 47, 51)	Irregular contractile activity
	Phase III		18 (7, 13, 15, 17, 18, 28, 29, 31, 33–38, 45–47, 50, 51)	Regular sustained contractions, with a minimum duration of 3 min, and minimum contraction frequency of 8 per minute
Discrete clustered contractions		Migrating clustered contractions (46), Propagating sequence (4, 26, 27), Type III and Type IV waves (20, 23, 24, 44)	12 (12, 18, 25, 26, 28, 29, 35, 39, 40, 45, 47, 50)	A group of phasic contractions, with maximum duration of 3 min, and contraction frequency up to 12 per minute
Prolonged propagated contraction		Propagated contractions (35), Large pressure waves (48)	14 (12, 15, 17, 18, 25, 29, 32, 39, 40, 42, 45–47, 50)	A single wave, with minimum duration of 6 s and minimum amplitude of 20 mmHg that propagates
Regular phasic contraction		Type I waves (20, 23, 24, 44)	2 (16, 22)	Wave peak with rate of pressure increase > 4 mmHg/sec, and lasting ≤ 8 s
Prolonged phasic contraction		Type II waves (20, 23, 24, 44)	2 (16, 22)	Wake peak with rate of pressure increase > 4 mmHg/sec, and lasting > 8 s
Slow phasic contraction			1 (16)	Wave peak with rate of pressure increase between 1 and 4 mmHg/sec

*MMC, Migrating motor complex*.

There were motility terms which only featured in a small number of studies. Examples included “Migrating Clustered Contractions” (46), “Propagated Contractions” (35), “Large Pressure Waves” (48), and “Propagating Sequence” (4, 26, 27). Their definitions were similar to the main motility terms listed above. Therefore, in the review analysis, they were considered together with the main terms, as shown in Table 2. Phasic contractions (and their subgroups) were entirely different patterns and were analyzed separately.

Four older studies used the terms type I, type II, type III and type IV waves (20, 23, 24, 44) which were later replaced by more modern names described above following the description of the MMC in 1969 (52). These older terms were analyzed together with their modern counterparts, as shown in Table 2.

Three studies defined the motility index as “log_e_(sum of amplitudes × number of contractions +1)” (30, 39, 40), one study described it as “log_10_(sum of amplitudes × number of peaks +1)” (32), four studies used the integration of the area under the pressure tracings (31, 34, 48, 49) and one defined it as “[sum of log(amplitudes of contractions)]/number of contractions” (36).

#### Interdigestive Motility in Healthy Individuals

The four most commonly reported motility patterns found during fasting were the MMC, DCCs, PPCs, and phasic contractions. A summary of their parameters is displayed in Table 3. Several researchers noted that these motility patterns were a small component of the total recording period and that the predominant activity appeared to be frequent irregular contractions (18, 45). Some found long periods of inactivity interspersed with more active periods (49).

**Table 3:** Summary of parameters for ileal manometry motor patterns in normal healthy individuals.

**Motility pattern**	**Incidence**	**Duration (min)**	**Amplitude (mmHg)**	**Frequency of contractions (no. per min)**	**% Propagated**	**Propagation velocity (cm/min)**	**Propagation distance (cm)**
Phase III of MMC	1 event every 1.3–40 hrs^#^ (7, 13, 15, 36, 37, 45, 46, 49, 50)	6.5–18.8^*^ (7, 13, 15, 31, 33, 37, 38, 45, 46, 50, 51)	27.9–39.4^*^ (28, 46, 51)	8.0–11.9^*^ (7, 15, 28, 33, 35, 37, 38, 45, 46)	29–90^#^^$^ (13, 45, 46, 49)	0.6–3.1^*^ (7, 13, 15, 28, 31, 33, 38, 45, 46, 50)	5.2–5.3^*^ (46)
Discrete clustered contractions	1 event every 0.03–33 hrs^#^ (25, 26, 28, 45, 46, 49, 50)	0.7–2.6^*^ (25, 45, 50)	No Data	8.7^*^ (22)	44^#^ (22)	13.8–120^*^ (22, 25, 45, 50)	50^#^ (45)
Prolonged propagated contraction	1 event every 0.33–17.1 hrs^#^ (13, 15, 21, 25, 33, 35, 45, 46)	0.2–0.3^*^ (15, 21)	42–60.2^*^ (15, 46)	No Data	No Data	27–60^*^ (21, 45)	20–50^#^ (33)
Regular phasic contraction	No Data	0.1^*^ (22)	27.9–32.1^*^ (22)	No Data	27.9^#^ (22)	18^*^ (22)	No Data
Prolonged phasic contraction	No Data	0.3–0.4^*^ (22)	28.4–47.3^*^ (22)	No Data	31.2^#^ (22)	24^*^ (22)	No Data
Slow phasic contraction	No Data	No Data	No Data	No Data	No Data	No Data	No Data

*MMC, Migrating motor complex*;

* *range of reported mean values*;

# *range of reported data values*.

$ *All phase III of the MMC must propagate by definition. These values represent the percentage of phase III activity that were seen to propagate based on the manometry techniques of the respective research teams*.

##### Migrating motor complex

The MMC (with its three phases) was an infrequent finding in the ileum. Almost half of the studies (twelve) did not present any data on phase III/MMC. One of these studies had a prolonged recording period of 24 h (25). Most studies detected one phase III every 1.3–3.2 h of recording. One researcher noted that the incidence decreased dramatically when recording occurred close to the ileocaecal junction with one phase III detected every 40 h in this region (45).

The mean value of the total duration of each MMC cycle in the ileum ranged from 90 to 134 min (38, 46, 50). Of the three MMC phases, phase II was found to be the longest with one study reporting a mean duration of 64 min (46). The mean duration of phase I ranged from 19 to 39 min (15, 46). Phase III was generally the shortest in duration, with mean values ranging from 6.5 to 15.6 min (15, 33). During sleep, two studies reported shorter or absent phase II (7, 37).

A single study reported contraction frequencies (mean of 540 waves per minute) and propagation velocities (mean of 78 cm/min) many orders of magnitude higher than the others (46). Given the significant discrepancy, this data was not included in the summary data table. Confirmation of data accuracy was sought via email correspondence but no response was received.

A small proportion of phase III patterns that were detected proximally in the small bowel propagated to the terminal ileum. Researchers found while 42% of MMCs propagated to the proximal ileum, only 4–9% propagated as far as the distal ileum (15, 33). An even smaller proportion (2%) progressed to the ileocaecal junction (45).

To investigate the motor-flow relationship, researchers infused a non-absorbable marker, phenolsulfonphthalein, via a recording orifice of the manometry catheter assembly and then aspirated the downstream fluid (a mixture of marker and bowel content) to calculate flow based on concentration differences. They found 50% of flow occurred during phase III, 30% occurred during phase II and 20% occurred during phase I (36). One research team found an association between phase III and ileocolonic bolus movement in only four out of nine patients (30).

##### Discrete clustered contractions

The majority of studies in healthy individuals (19 out of 28) did not present any data on DCCs or a similar pattern. When data was presented, the incidence was variable. Four studies found one DCC every 0.03–0.2 h, making them much more common than the MMC in the ileum (26, 45, 46, 49). However, two studies found them to be infrequent with one DCC detected every 4–33 h (25, 28).

Discrete clustered contractions appeared to have a limited relationship with colonic motility. One study found that 15% of DCCs were associated with caecal propagating sequences while another found no relationship between DCCs and colonic filling (25, 30). This motor pattern was also found to have a limited role in propulsion of ileal contents, with one researcher detecting no consistent association with caudal propulsion of an isotope (49).

No studies reported data on the amplitude of DCCs in healthy individuals.

##### Prolonged propagated contractions

Prolonged propagated contractions were infrequent in the ileum. Twenty studies out of 28 did not present any data on PPCs or a similar pattern. One study that failed to identify this pattern had a prolonged recording period of 36 h (49). The majority of the studies which did detect PPCs found one event every 3.3–17.1 h (21, 46). Only one report found a higher incidence in some of their study participants, detecting one event every 0.33 h (45).

This motility pattern appeared to be more common during sleep, as one study found the incidence of propagating events (including PPCs and DCCs) were three times higher during the nocturnal period compared to day time (25).

The association between PPCs and caecal motility was greater than DCCs. One researcher found 46% of PPCs was associated with caecal propagating sequences (25). No studies reported data on the percentage of PPCs that were propagated.

##### Phasic contractions

Similar to PPCs, phasic contractions had some relationship with caecal motility. One study found 36.7 and 41% of regular phasic and prolonged phasic contractions, respectively, were associated with caecal propagating sequences (22). They also found 13.3 and 29.2% of regular and prolonged phasic contraction, respectively, directly propagated from the ileum to the caecum.

##### Motility index

Based on the log definition of the motility index, one researcher found the mean motility index to range from 10.11 to 10.45 (30). Those that used the area under the pressure tracing found mean values that ranged from 6.1 to 17.4 mmHg.min (31, 48, 49).

#### Post-prandial Motility in Healthy Individuals

After ingestion of food, most researchers found replacement of the interdigestive motility patterns, such as the MMC, with intense contractions that appeared irregular and failed to meet the definition criteria of any known motility patterns (12, 33, 37, 45, 49, 50). There was also an absence of other fasting patterns such as DCCs and PPCs (45, 50). If an MMC was detected at the start of the meal, eating interrupted further progression of that MMC (7). The onset of this post-prandial pattern was as rapid as 10 min after meal ingestion (50). The duration of this response ranged from 2.2 to 10.5 h (7, 13, 35, 37, 46). The mean motility index was significantly higher following food ingestion, with a mean value (SEM) (based on area under pressure tracings) of 23.3 ± 2.0 mmHg.min (49). This increase was sustained for only 30 min before returning to fasting levels.

This abolition of interdigestive motility patterns was not found by all researchers. One study found the fasting pattern continued in 4/6 patients in one series and 5/7 in another (30). Another study found a single instance of phase III-like activity 42 min after a meal in one patient (37). DCC-like patterns were also noted during the post-prandial period, with one researcher reporting an incidence of one event every 13 min (27, 46). The mean (SD) amplitude of the DCC-like pattern was reported to be 22.9 ± 9 mmHg (27) and the mean (SEM) propagation velocity was 84 ± 6 cm/min (46).

#### Ileocaecal Junction in Healthy Individuals

Only two studies investigated the motility patterns of the ileocaecal junction in normal individuals (22, 45). One researcher used an incremental pull-through technique of the manometry catheter across the ICJ introduced via colonoscopy (22) while the other used prolonged naso-enteric intubation with a perfused manometry catheter that had sensors which straddled the ICJ (45).

Both of these studies failed to identify a sustained high-pressure zone in the ICJ region. One researcher found elevation of tonic pressure that was detected only during periods of sustained phasic contractions which were present less than 10% of the recording time (45). The range of pressures found was 0–34 mmHg. However, not all phasic contractions elicited increase in tonic pressures at the ICJ.

#### Motility in Individuals With Gastrointestinal Disorders

Five studies reported manometry findings in patients with motility disorders, including chronic idiopathic constipation (13), irritable bowel syndrome (12, 28, 35) and acute salmonellosis (15). They key similarities and differences of the manometry findings from these patients compared to normal individuals are summarized in Table 4.

**Table 4:** Summary of similarities and differences in ileal manometry features in motility disorders.

	**Similarity/difference**			
	**Fasting manometry features**			**Post-prandial manometry features**
**Motility disorder**	**MMC**	**DCC**	**PPC**	
Chronic idiopathic constipation	Similarities: Phase III: Incidence (1 every 3.4 h)^$^ and propagation velocity (0.9 ± 0.4 cm/min)^#^ (13) Differences: Phase II: Longer duration (54.1 ± 8.5% of total recording period vs. 8.2 ± 2.1% in controls)^#^ ^*^ (13) Phase III: Shorter duration (7.9 ± 1.8mins vs. 13.2 ± 1.3 min in controls)^#^ ^*^ (13) Phase III: Smaller percentage propagating (62 vs. 90%)^$^ (13) Phase III: Retrograde propagation more common (11 instances vs. none in control group)^$^ (13)	No Data	Differences: Incidence: Lower (1 event every 12 h vs. 1 even every 4 h in controls)^#^^*^ (13)	Differences: Duration of response: Shorter (30.1 ± 6.2 min vs. 130 ± 68 min in controls)^#^^*^ (13)
Irritable bowel syndrome	Similarities: Phase III: Duration, frequency of contractions (7.7–10.1 per minute)^&^, amplitude (41.6 ± 1.5 mmHg)^#^, propagation velocity (2.9 ± 0.2 cm/min)^#^ (28) Differences: Phase III: Higher proportion migrating to distal ileum (26 vs. 9% in controls)^$^ (35)	No Data	Similarities: Incidence (1 every 3.6–8.8 h)^$^ (35)	Similarities: Duration of response (12, 35) Replacement of MMCs with random, irregular contractions (12, 35)
Acute salmonellosis	Similarities: Phase III: Duration (7.5 ± 2.3 min)^#^, propagation velocity (2.5 ± 1.3 cm/min)^#^, and percentage propagating into ileum (37%)^$^ (15) Differences: Phase III: Lower incidence (1 event every 5.5 h vs. 1 event every 1.6 h)^$^ (15)	No Data	Similarities: Duration (0.23 ± 0.03 min)^#^ and amplitude (48 ± 4 mmHg)^#^ (15) Differences: Incidence: Higher (1 event every 1.3 h vs. 1 event every 6.7 h)^$^ (15) Propagation velocity: Higher (mean 156 cm/min) (15)	No Data

*MMC, Migrating Motor Complex; PPC, Prolonged Propagated Contraction; DCC, Discrete Clustered Contractions*;

# *mean ± SEM*.

* *Statistically significant difference (p < 0.05)*,

$ *reported values*,

& *range of reported means*.

##### Irritable bowel syndrome

One study only found a significant difference in frequency of contractions of phase III in the distal ileum (7.7 ± 0.2 vs. 8.5 ± 0.2) but not in the proximal ileum (35). Discrete clustered contractions were noted to be infrequent in the ileum of IBS patients but no specific data were presented (28). There was suggestion that prolonged propagated contractions may be associated with abdominal pain. One study administered cholecystokinin octapeptide infusions to stimulate the small bowel and the PPCs generated were concurrent with pain in 4/5 patients with constipation-predominant IBS (12). Neostigmine injections were also given for the same purpose and the PPCs generated were concurrent with symptoms in all 7 IBS patients. Another research team found that 61% of PPCs were directly related in time to cramping discomfort in IBS patients compared to 17% in healthy controls (35). Cramping was rare in the absence of PPCs. There was no correlation between the discomfort and the magnitude of the pressure peak or propagation to the caecum.

It was unclear if this association between PPCs and abdominal discomfort was limited to IBS patients as researchers also found 4/5 healthy volunteers suffered symptoms concurrently with PPCs (12). Other studies found no association between episodes of pain and any small intestinal motility patterns (28).

##### Acute salmonellosis

Researchers commented on the presence of silent periods which were not found in healthy controls. This was defined as episodes of motor quiescence (with fewer than three waves over 12 mmHg in a 10-min period) simultaneously recorded over two consecutive manometry sites that lasted for more than 20 min but did not fulfil the criteria for phase I as it was not preceded by phase III (15). These periods had a mean duration [SEM] of 64 ± 32 mins and affected half the length of the ileum.

#### Motility in Individuals After Bowel Surgery

Ten studies investigated ileal motility through either a loop or end ileostomy (4, 16, 20, 23, 24, 39–41, 44, 47). Five studies assessed the terminal ileum adjacent to an ileal pouch formed after proctocolectomy (17, 29, 39, 43, 50). Two studies featured patients who had colonic exclusion for management of hepatic encephalopathy (3, 19) with one of these studies also featuring a patient with double-barrelled ascending colostomy for management of a pancreatic fistula (3). The key similarities and differences in manometry features compared to normal individuals are shown in Table 5.

**Table 5:** Summary of similarities and differences in ileal manometry features in bowel surgery patients.

	**Similarity/difference**			
	**Fasting manometry features**			**Post-prandial manometry features**
**Surgical procedure**	**MMC**	**DCC**	**PPC**	
Ileostomy	Similarities: Phase III: Frequency of contractions (8 per minute)^#^ and propagation velocity (2.5 cm/min)^#^ (47)	Similarities: Incidence (1 every 0.2–2.5 h)^#^^$^ and propagation velocity (11.7 cm/min)^$^ (4, 39, 40) Differences: Duration: Shorter (0.1 to 1.5 min)^$^ (4, 20) Percentage propagated: Lower (28%)^#^ (39) Retrograde DCCs (46% of DCCs)^#^ (39)	Similarities: Incidence (1 every 0.9–1.7 h)^#^ (39, 40)	Similarities: Increase in active contractions and motor activity (47) DCC: Incidence (1 every 0.02–2 h)^#^^$^, duration (0.8–1.2 min)^#^^$^ and amplitude (16.1–28.7 mmHg)^#^^$^ (4, 20, 27, 39, 40) PPC: Incidence (1 every 0.59 h)^$^ (40)Differences: Some studies found no change in motility index after food (39, 40) Duration of post-prandial response: Lower (60–90 min)^#^ (4, 23, 44) DCC: Percentage propagated: Higher (up to 59.1%)^$^ (39, 40)
Proctocolectomy with Ileo-anal Pouch	Similarities: Phase III: Incidence during daytime in patients with poor pouch function (1 every 1.88 h)^&^^*^ (29) Phase III: Duration (6–10 min) ^$^^&^ (29, 50) Phase III: Tend to fade out in terminal ileum (29, 50) Differences: Phase I: Duration: Longer (49–80 min)^&^ (29) Phase III: Incidence in evening: Higher in patients with poor pouch function^*^ (1 event every 0.88 h)^&^ (29) Phase III: Propagation velocity: Higher (up to 7 cm/min)^&^ (29, 50)	Similarities: Duration (0.7 min)^$^ and propagation velocity (116 cm/min)^$^ (50) Differences: Incidence: Higher in patients with poor pouch function (1 event every 0.5 h)^&^ (29)	Similarities: Incidence (1 every 0.08–2.7 h)^#^ (17, 50)	Similarities: Intense, random contractions (50) Duration of response (73–360 min)^#^ (29, 50) Rapid onset of response (within 10 min)^#^ (50) Absence of PPCs and DCCs (50) Differences: Persistence of fasting motility pattern in one study (17)

*MMC, Migrating Motor Complex; PPC, Prolonged Propagated Contraction; DCC, Discrete Clustered Contraction*;

* *Poor pouch function defined as more than 10 bowel movements in 24 h*,

# *Reported values*,

$ *Reported mean values*,

& *Reported median values*.

##### Ileostomy

Three studies utilized manometry in only the afferent limb of a loop ileostomy (20, 23, 24), four studies only investigated the efferent limb (16, 39–41) and one research team measured motility in both limbs (4). No direct comparisons between motility of the afferent and efferent limbs were made. One study featured end ileostomies (16) while two studies did not specify which limb was assessed (44, 47).

Only one study identified phase III complexes, which included a pediatric population only (47). Their predominant finding was that of irregular, intermittent contractions. The characteristics of the detected motility patterns were similar to those found in normal adults.

The duration of regular and prolonged phasic contractions were similar (16). The mean amplitudes of regular and prolonged phasic contractions (36–37 and 58–59 mmHg, respectively) were higher than the values seen in normal individuals (16). There appeared to be a smaller percentage of regular phasic contractions that propagated (4–12.5%). The propagation velocity of regular phasic contractions appeared to be higher, with a mean of 30 cm/min. The motility index, based on the log definition, appeared slightly lower with mean values ranging from 8.1 to 10.0 (39, 40).

Researchers reported data for which there were no corresponding values in normal individuals to allow comparison. This included the mean amplitude of DCC [15.5–68.0 mmHg (4, 39)], the percentage of PPCs found to propagate [85% (39, 40)], and the mean frequency of contractions of regular phasic contractions [7.0–8.6 per minute (16, 20)]. Slow phasic contractions were found to have a mean duration of 18–20 s, a mean amplitude of 23–24 mmHg and 6–20% of contractions were found to propagate (16). The same study also showed 36.7–64.3% of phasic contractions were associated with caecal motor activity.

Three studies used the ileostomy as an access point for investigating the ICJ (4, 16, 41). There were inconsistent findings. One researcher was able to identify a region of sustained increased tone with a mean (SEM) pressure of 9.7 ± 3.2 mmHg and a mean length of 4.8 cm (4). However, one study failed to identify a discrete high-pressure zone while another only detected an increase in tone associated with increased phasic activity at the ICJ (16, 41). This phasic activity had a frequency of contraction of 4–8 per minute and was present during a mean of 35% of fasting recording time (4). Ileal propagating wave patterns had primarily an inhibitory effect on phasic and tonic activity at the ICJ and all such wave patterns which extended through the ICJ to the caecum did so only when phasic activity was absent in the region (4).

The effects of ileal and colonic distension on ICJ tone were also investigated. Colonic distension produced an increase in tone (mean of 2.8–3.5 mmHg). This increase affected both the afferent and efferent ileal limbs (4). However, ileal distension produced mixed results with one researcher finding a mean decrease in ICJ tone of 2.8 mmHg, while another found instances of increased, decreased and unchanged ICJ tone (4, 41). The effects of distension on phasic activity were also noted. Colonic distension produced either an increase (mean difference of 7.4 mmHg) or no change to the amplitude of phasic waves. The duration of phasic activity also increased from a mean of 17% of total recording period to 55% (41). Ileal distension produced opposite effects, with a mean decrease in phasic wave amplitude of 5.9 mmHg and a decrease in the duration from 60 to 15% of the recording time. This was not the case in all instances as 4% of ileal distension episodes produced an increase in phasic activity and 20% of distensions produced no change (41).

In the post-prandial period, one research team noted an immediate increase in ICJ tone with a mean pressure of 11.8 mmHg that was sustained for 90 min. Following this time period, the tone reduced down to a mean of 4.9 mmHg, which was lower than the fasting value (4). After food ingestion, retrograde propagation was found in 41–50% of propagated DCCs. One researcher noted the amplitude of regular phasic contractions increased after eating (24). The amplitude of prolonged phasic contractions appeared to be lower (11.0 mmHg) than fasting values (24). The frequency of contractions of regular phasic contractions (6.6–7.9 per minute) was slightly lower than fasting values in normal individuals (20).

##### Proctocolectomy

Five studies investigated patients with proctocolectomy with ileal-anal anastomosis. The specific procedures performed were ileal pouch-anal canal anastomosis with diverting loop ileostomy (39), ileal pouch-anal canal anastomosis after reversal of the loop ileostomy (29, 50), ileal pouch-anal canal anastomosis with no specific history of diverting ileostomy (17) and pelvic ileoanal reservoir with a short efferent ileal limb between the pouch and anal canal (43).

One study failed to identify phase III (17). The duration of phase II was found to have a median value of 4–7 min. This parameter was not reported in studies of normal individuals (29). Discrete clustered contractions were reported to be the dominant pattern in the ileum in one study, with the majority being non-migratory (39). There was a particularly high incidence of PPCs in patients who complained of frequent stools, with one event every 0.08–0.2 h (17). The range of PPC amplitude values found were between 50 and 100 mmHg (17). After food ingestion, DCCs were only found in the late post-prandial period beyond 3 h (50).

Motility of the ileal pouch itself was characterized by irregular sporadic contractions (17). One study noted <2% of all phase III complexes and none of the DCCs migrated into the pouch reservoir (29). In contrast, multiple PPCs were found to propagate into the pouch (29). The timing of bowel movements and the sensation of urge to defecate did not correlate with any motility pattern (29, 50). Defecation occurred in all phases of the MMC. Other researchers noted two patterns of motor activity within the pouch: short waves 2–6 s in duration and long waves up to 30 s (43). After ingestion of food, one research team noted a two-stage effect: disorganization of phase III followed by large propulsive contractions, sometimes causing urgency (43).

Two studies investigated the effect of pouch inflation on the motility of the pouch and proximal ileum. One study found pouch distension up to functional capacity produced polyphasic pressure waves with contractions lasting 15–25 s, with amplitudes of 14.7–22.1 mmHg in the pouch and 44.1–51.5 mmHg in distal limb (43). The other study found no change in motility patterns (29).

One research team focused on motility differences between patients with good pouch function and those with poor pouch function, defined by the number of bowel movements per day (29). Patients with poor function had a significantly longer period of increased activity prior to defecation (25 vs. 9 min, *p* = 0.003), a higher incidence of the MMC at night, and overall shorter duration of motor quiescence (phase I). All other parameters including duration of MMC, incidence of MMC during the day time, incidence of DCCs and PPCs and the duration of the post-prandial response were similar. Another study found that ileal pouch patients with excessive stools had frequent non-propagated high amplitude contractions within the pouch that was often accompanied by defecation (17).

##### Colonic exclusion

Two studies evaluated patients who had undergone colonic exclusion procedures. In both studies, the procedure left patients with an ileostomy in the right lower quadrant (3, 19). One researcher also included a patient with a double-barrelled ascending colostomy (3). These investigations focused on the ICJ region only.

Both of these studies found a discrete high-pressure zone at the ICJ with a length of 4 cm and a mean pressure of 20.3–21 mmHg (3, 19). Distension of the caecum was found to cause a mean increase in the ICJ tone of 17 mmHg while distension of the ileum caused a mean decrease of 12 mmHg (3). These effects were present during 80 and 87% of colonic and ileal distension episodes, respectively. The change in tone occurred rapidly after a mean of 2.3 s following distension and the duration of the change was correlated directly with the length of balloon distension.

## Discussion

### Summary of Main Findings

This study reports a systematic review summarizing the current knowledge of human ileum and ICJ manometry findings in the context of health, motility disorders or after bowel surgery during both fasting and fed states. The motility in health demonstrated distinct patterns during fasting compared to the post-prandial period. While fasted, the main patterns found were the MMC and its three phases, DCCs, PPCs, and phasic contractions. DCCs appeared to be the most common with MMC phase III being the rarest. However, the incidences of these patterns were highly variable (as per Table 3). Phase III of the MMC had the longest duration lasting for several minutes, whereas DCCs, PPCs and phasic contractions often lasted <1 min. A large proportion of phase III activity appeared to propagate (based on the available manometry techniques) but at slower velocities compared to the other patterns. Almost half of all DCCs propagated while less than a third of regular and prolonged phasic contractions propagated. Prolonged propagated contractions appeared to have the highest amplitude of any pattern. During the post-prandial period, these patterns became much harder to detect and the predominant finding was intense, irregular contractions, which persisted for 3–4 h.

The functional significance of these patterns has been evaluated and theorized. Phase III of the MMC is thought to clear the luminal contents and act as the intestinal “housekeeper” (53). As PPCs were found to be triggered by infusing short chain fatty acids into the ileum, they may protect against coloileal reflux (32). Findings from animal studies support these theories. Each passage of phase III of the MMC in the canine ileum is associated with emptying of half of luminal contents into the colon and instillation of short chain fatty acids into the ileum of animal models also triggered PPCs which cleared refluxate from the colon (54–57). However, given the rarity and often complete absence of these two motility patterns in the human ileum, their importance in this region is unclear.

There were inconsistent findings regarding the ICJ. Some researchers found a 4 cm segment of sustained increased tonic pressure while others did not (22, 45). When such a segment was found, the mean pressure detected ranged from 9.7 to 21 mmHg. The pressure of the ICJ region was comprised of both a tonic component as well as phasic activity, present for up to 35% of the recording period (4). Experimental distension of the colon tended to produce an increase in the ICJ tone while distension of the ileum produced a decrease in ICJ tone, but this was inconsistent. The variable response of the ICJ to colonic distension may partially explain why up to 25% of the population are thought to have an incompetent ileocaecal valve but further targeted studies are required to confirm this (58).

Although studies were limited, manometry findings in patients with motility disorders differed from healthy controls in a few aspects. Patients with chronic idiopathic constipation had longer duration of phase II and phase III, lower incidence of PPCs, and shorter duration of post-prandial response (13). Patients with IBS had significantly lower frequency of contractions of phase III and PPCs tended to have a stronger association with abdominal pain symptoms, although this was inconsistent. Acute Salmonellosis patients had “silent periods” with little motor activity that lasted for more than an hour which was not seen in healthy controls (15).

Bowel surgery patients also demonstrated differences in manometry findings compared to healthy controls. In patients with an ileostomy, researchers found retrograde DCCs, higher amplitude of regular and prolonged phasic contractions, and higher propagation velocity of regular phasic contractions. However, none of the ileostomy studies were case controlled. Data collected exclusively from the efferent limb did not reveal any notable differences compared to data from the afferent limb between the studies. Only one researcher placed manometry catheters into both the afferent and efferent limbs of a loop ileostomy in the same patient but no direct comparisons were made (4).

Patients who had proctocolectomy with ileal pouches showed a longer duration of phase I compared to healthy controls. Comparison between those with good pouch function and poor function showed a significantly longer period of increased activity prior to defecation, higher incidence of MMC at night and shorter duration of phase I in those with poor function. Studies involving colonic exclusion patients focused on the ICJ only and identified a discrete high-pressure zone of 4 cm in length.

The motility characteristics of healthy individuals exhibited significant variability and this wide variation appears to limit the ability of ileal manometry to readily distinguish pathological motility from normal motility. Analysis of data from patients with motility disorders, e.g., IBS, and patients who underwent bowel surgery, e.g., loop ileostomy, in this systematic review found few major differences in motility patterns. This variability may be a true intrinsic feature of human ileal motility but two factors that may confound interpretations are the difficulties of manometry equipment and the lack of standard definitions.

### Limitations

Accurate assessment of the terminal ileum using manometry is challenging. As shown in Table 1, researchers employed a variety of manometry techniques with different catheter specifications and protocols. Each of these details could potentially influence motility measurement. For example, a stiff manometry catheter with a large diameter could lead to higher pressures recorded (4, 46). Large spacing, e.g., 20 cm, between sensors could reduce the sensitivity for detecting propagation, which may lead to some instances of phase III with short migration after reaching the ileum being missed or wrongly classified (15). A long manometry catheter introduced via oral or nasal route could induce sleeving of the small intestine over the catheter which would underestimate propagation velocities (28). The viscosity of luminal contents can also alter manometry measurements (59). Short recording durations may fail to detect motility patterns due to their low incidence (47). A low caloric content of the ingested meal during the experiments may not be sufficient to trigger a motility response in the ileum (30). Restrictions placed on study participants during recording, e.g., limitations on mobility, prolonged starvation, and being in a foreign environment, may induce stress which is known to affect small bowel motility, especially in IBS patients (28).

Manometry assessments of an ileal pouch could be confounded by the pouch construction itself. When walls of large-volume, low-pressure reservoirs contract, this may not translate to an accurate rise in intra-luminal pressure detected by manometry. This limitation could account for the lower motility index and fewer cluster contractions detected in the included studies (40).

The ileocaecal junction is relatively inaccessible and dynamic, creating difficulties in precise positioning of manometry sensors. For example, a long nasoenteric manometry catheter with widely spaced sensors does not allow for the accurate placement and maintenance of a sensor within the relatively short segment of the ICJ. Using bowel preparation to allow placement of a manometry catheter via colonoscopy as well as the caecal distension (which studies in this review has shown could alter the ICJ pressure profile) from the colonoscope could also confound ICJ motility findings (22, 41, 45). However, one researcher directly compared ICJ recording using endoscopy to a trans-ileostomy approach and found similar results (16).

A significant problem is the lack of accepted standard definitions of ileal motility patterns. Up to 18 studies described phase III, DCCs and PPCs and there was little agreement of the defining characteristics. The low incidence of these patterns reported in some studies may be due to strict definition criteria that the observed manometry patterns failed to meet. The same manometry findings may have fulfilled the criteria for a particular term of a different research team. Therefore, inconsistent definitions weaken the ability to make meaningful comparisons across studies.

Accurate assessment of ileal motility may be of benefit to patients who have undergone bowel surgery, such as loop ileostomy. Common complications following ileostomy reversal include small bowel obstruction and prolonged post-operative ileus with rates as high as 20% (60). Histology studies have demonstrated villous atrophy and reduction in the strength and duration of circular smooth muscle contractions in the distal ileal limb following a period of faecal diversion (61–63). It is plausible that this loss of contractility may lead to a “functional” obstruction at time of ileostomy reversal resulting in the aforementioned complications. The loop ileostomy is thus not only an easy access point but also an area where greater understanding of motility could inform improved post-operative outcomes. No studies found in this review directly compared efferent limb motility to healthy controls or to the afferent limb. Manometry studies involving such comparisons may help to assess the effects of faecal diversion on bowel motility.

Future research into ileal motility must attempt to optimize elements of manometry technique and study protocol. Recording should occur over a prolonged period of fasting to allow detection of infrequent patterns, i.e., at least 3 h. If prolonged day-time recording is planned, eating three meals a day will likely lead to absence of motility phenomena given the abolition of the MMC in the post-prandial period. Reducing the inter-sensor spacing would provide better characterization of pattern propagation (64). When assessing the ICJ, effort should be made to accurately confirm sensor placement and utilization of ileostomies could be a safe, convenient and effective method. Studies should strive to use manometry pattern definitions either from extant literature with similar investigative focus or the summary definitions in this review to provide better consistency and avoid creating further new definitions.

Contradictory data exists regarding the effect of food ingestion on fasting motility, the presence or absence of a discrete high-pressure zone at the ICJ, the effect on the ICJ of ileal distension and associations between PPCs and abdominal discomfort in IBS patients. Further studies focused on addressing these inconclusive aspects of ileal motility whilst being mindful of the above measurement and protocol issues could be beneficial.

Consideration should be given to the establishment of an international and interdisciplinary consensus group to help formulate standardized operation protocols. Manometry pattern definitions can also be agreed upon and such a group could also assist with co-ordination of a multi-center approach to recruitment and data collection to increase study power.

### Conclusions

Manometry recording of the terminal ileum in health shows different motility patterns during fasting compared to the post-prandial period. There exists large variability of pattern characteristics in health. The exact motility pattern of the ileocaecal junction is inconclusive with conflicting findings. Motility patterns show some differences in patients with motility disorders and bowel surgery, however technical challenges and lack of standardized definitions impair researchers' abilities to accurately assess this critical region of the gastrointestinal tract. Future research is warranted with careful attention to the influence of manometry technique and study protocol.

## Data Availability Statement

The raw data supporting the conclusions of this article will be made available by authors, without undue reservation, to any qualified researchers.

## Author Contributions

CL, IB, and GO'G contributed to the design of the protocol. CL and KS performed the literature search. CL extracted the data. KS checked data accuracy. CL, GO'G, PD, and IB contributed toward the writing and editing of the manuscript.

### Conflict of Interest

The authors declare that the research was conducted in the absence of any commercial or financial relationships that could be construed as a potential conflict of interest.
